# How is the McGurk effect modulated by Cued Speech in deaf and hearing adults?

**DOI:** 10.3389/fpsyg.2014.00416

**Published:** 2014-05-19

**Authors:** Clémence Bayard, Cécile Colin, Jacqueline Leybaert

**Affiliations:** Center for Research in Cognition and Neurosciences, Université Libre de BruxellesBrussels, Belgium

**Keywords:** multimodal speech perception, Cued Speech, cochlear implant, deafness, audio-visual speech integration

## Abstract

Speech perception for both hearing and deaf people involves an integrative process between auditory and lip-reading information. In order to disambiguate information from lips, manual cues from Cued Speech may be added. Cued Speech (CS) is a system of manual aids developed to help deaf people to clearly and completely understand speech visually (Cornett, 1967). Within this system, both labial and manual information, as lone input sources, remain ambiguous. Perceivers, therefore, have to combine both types of information in order to get one coherent percept. In this study, we examined how audio-visual (AV) integration is affected by the presence of manual cues and on which form of information (auditory, labial or manual) the CS receptors primarily rely. To address this issue, we designed a unique experiment that implemented the use of AV McGurk stimuli (audio /pa/ and lip-reading /ka/) which were produced with or without manual cues. The manual cue was congruent with either auditory information, lip information or the expected fusion. Participants were asked to repeat the perceived syllable aloud. Their responses were then classified into four categories: audio (when the response was /pa/), lip-reading (when the response was /ka/), fusion (when the response was /ta/) and other (when the response was something other than /pa/, /ka/ or /ta/). Data were collected from hearing impaired individuals who were experts in CS (all of which had either cochlear implants or binaural hearing aids; *N* = 8), hearing-individuals who were experts in CS (*N* = 14) and hearing-individuals who were completely naïve of CS (*N* = 15). Results confirmed that, like hearing-people, deaf people can merge auditory and lip-reading information into a single unified percept. Without manual cues, McGurk stimuli induced the same percentage of fusion responses in both groups. Results also suggest that manual cues can modify the AV integration and that their impact differs between hearing and deaf people.

## Introduction

In face-to-face communication, speech perception is a multi-modal process involving mainly auditory and visual (lip-reading) modalities (Sumby and Pollack, 1954; Grant and Seitz, 2000). Hearing-people merge auditory and visual information into a unified percept, a mechanism called audio-visual integration (AV integration). This merging of information has been demonstrated through the McGurk effect (McGurk and MacDonald, 1976), in which integration occurs even when auditory and visual modalities provide incongruent information. For example, the simultaneous presentation of the visual velar /ka/ and auditory bilabial /pa/ normally leads hearing-individuals to perceive the illusory *fusion* alveo-dental /ta/. The McGurk effect suggests that visual articulatory cues about place of articulation are integrated into the auditory percept which is then modified.

Presently, many children born deaf are fitted with cochlear implants (CI). This technology improves a child's ability to access auditory information. Studies have shown that deaf individuals (both adults and children) whom of which were fitted with CI's were able to integrate auditory and visual information, with better performance in the AV condition than in the audio condition (Erber, 1972; Tyler et al., 1977; Hack and Erber, 1982; Lachs et al., 2001; Geers et al., 2003; Bergeson et al., 2005; Desai et al., 2008). However, auditory information provided by the CI was degraded with respect to place of articulation, voicing and nasality (Dowell et al., 1982; Skinner et al., 1999; Kiefer et al., 2001). Therefore, participants fitted with a CI gave more importance to lip-read information in AV speech integration than did hearing participants (Schorr et al., 2005). In the case of incongruent auditory and visual information (McGurk stimuli), deaf participants (adults and children) gave more responses based on visual information, whereas hearing participants gave more integration responses or responses based on auditory information (Leybaert and Colin, 2007; Desai et al., 2008; Rouger et al., 2008; Huyse et al., 2013). However, the reliance on lip-reading information was flexible: when visual information was degraded, children with CI's relied less on visual information, and more on auditory information (Huyse et al., 2013). The AV integration is thus an adaptive process in which the respective weights of each modality depend on the level of uncertainty in auditory and visual signals.

**Figure 1:**
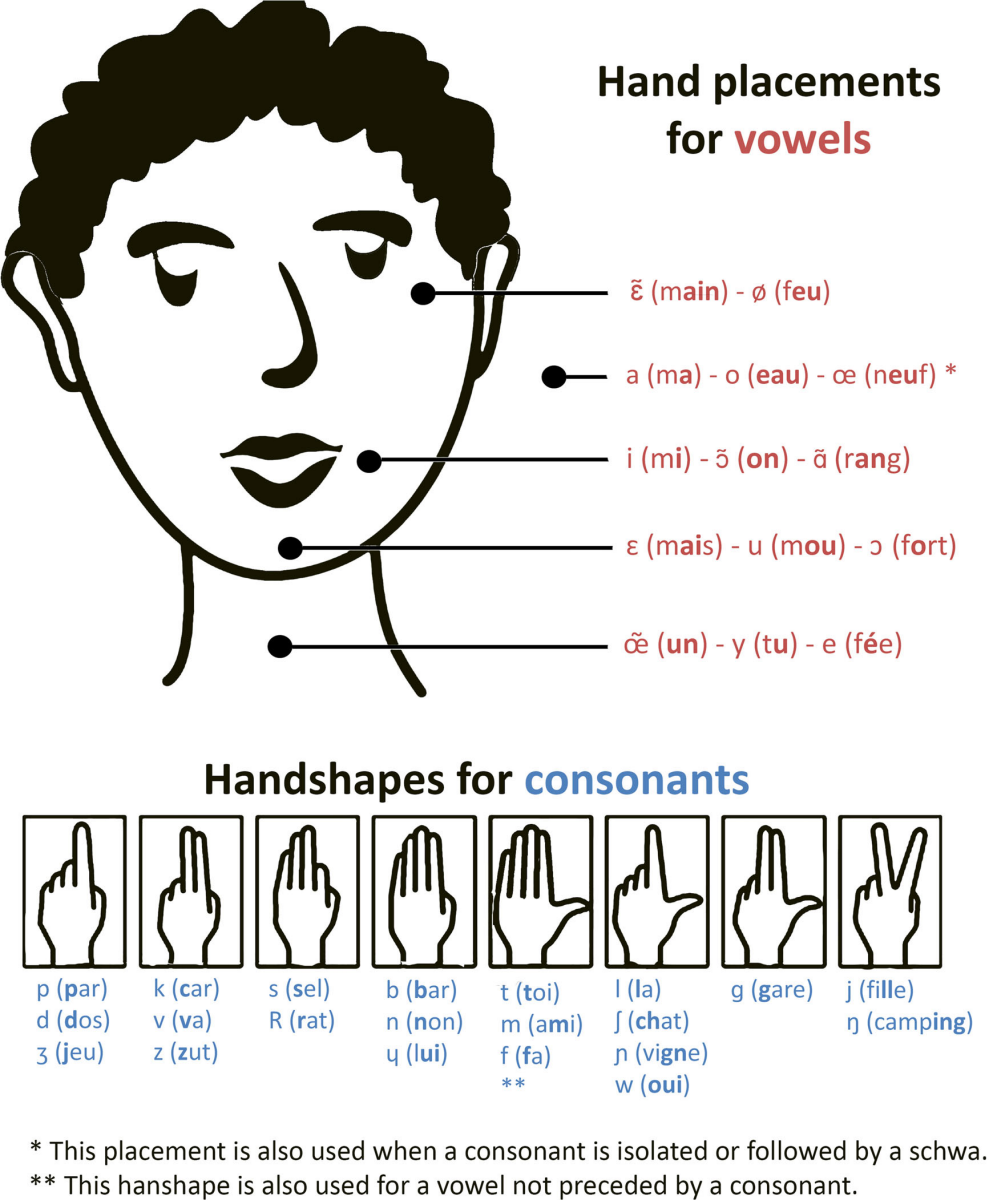
**Cues in French Cued Speech: hand-shapes for consonants and hand placements for vowels**. Adapted from http://sourdsressources.wordpress.com.

Aside from lip-reading, Cued Speech could help deaf people overcome the uncertainty of auditory signals delivered by the CI. Originally, the Cued Speech (CS) system was designed to help deaf people (without a CI) perceive speech through disambiguating the visual modality (Cornett, 1967). The CS system reduces the ambiguity related to lip-reading by making each of the phonological contrasts of oral language visible. Each syllable is uttered with a complementary gesture called a manual cue. CS was adapted to the French language in 1977, and is currently known as “Langue française Parlée Complétée.” In French, the vowels are coded with five different hand placements near the face, and consonants are coded with eight hand-shapes (see Figure 1). Each manual cue can code several phonemes, but these phonemes differ in their labial image. Also, consonants and vowels sharing the same labial image are coded by different cues. Thus, the combination of visual information, provided by the articulatory labial movements and manual cues, allows deaf individuals to correctly perceive all syllables. Nicholls and Ling (1982) studied the benefits of CS on speech perception. They compared deaf children's speech perception with or without CS and showed that the addition of CS improves speech perception from 30 to 40% in a lip-reading-only condition to 80% with the addition of manual cues. Similar results were found with French CS (Périer et al., 1990). Exposure to CS contributes to the elaboration of phonological representations, hence improving abilities notably in rhyme judgments, rhyme generation, spelling production as well as reading (Charlier and Leybaert, 2000; Leybaert, 2000; LaSasso et al., 2003; Colin et al., 2007).

While the advantages of exposure to CS are well-recognized, the processing of the CS signal still remains unclear. Attina et al. (2004) were the first to examine the precise temporal organization of the CS production of syllables, words, and sentences. They found that manual cues naturally anticipate lip gestures, with a maximum duration of 200 ms before the onset of the corresponding acoustic signal. In a second study, the same authors showed a propensity in deaf people to anticipate manual cues over lip cues during CS perception. That is to say, deaf people extract phonological information when a manual cue is produced whether or not lip movements are completed. This phonological extraction has the effect of reducing the potential number of syllables that could be perceived (Attina, 2005; Aboutabit, 2007; Troille et al., 2007; Troille, 2009). These results reverse the classic way of considering the CS system: manual cues, as opposed to labial information, could be the primary source of phonological information for deaf CS-users. Despite the fact that manual cues are artificial, they might constitute the main source of phonological information, and labial information would then be used to disambiguate this manual information.

Alegria and Lechat (2005) studied the integration of articulatory movements in CS perception. More precisely, they investigated the relative influence of labial and manual information on speech perception. Deaf children (mean age: 9 years, with normal intelligence and schooling) were split into two groups depending on their age of exposure to CS (early or late). They were asked to identify CV syllables uttered without manual cues (lip-reading alone) or with manual cues (Cued Speech). In the CS condition, lip movements and manual cues were either congruent (e.g., lip-reading /ka/ and hand-shape n°2, that codes /v, z, k/) or incongruent (e.g., lip-reading /ka/ and hand-shape n°1, that codes /d, p, Ʒ/). Identification scores were better in the congruent and lip-reading alone condition than when syllables were presented with incongruent manual cues. In the incongruent condition, participants reported syllables coded with the same manual cues as the actual syllables. Between the different syllables coded by a matching manual cue, deaf participants selected the one that had less visible lip movements; that is, the one that was less inconsistent with lip information presented in the syllable stimuli. For example, the lip movements /ka/ with hand-shape n°1 (coding /d, p, Ʒ/) was perceived as /da/ which is less visible on the lips than /pa/ and /Ʒa/. This suggests an integrative process between lip and manual cue information. Moreover, deaf children who were exposed to CS early (prior to 2 years) integrated manual cue and lip-read information better than deaf children who were exposed to CS later (after 2 years). To conclude, when lip-read information and manual cues diverge, participants choose a compromise that is compatible with manual information and not incompatible with the lip-read one.

The goal of the present research was to examine how manual cue information is integrated in AV speech perception by deaf and hearing participants. We wondered whether (1) CS receptors combine auditory, lips and manual information to produce a unitary percept; (2) on which information (auditory, labial or manual) they primarily rely; and (3) how this integration is modulated by auditory status. To address these issues we designed the first experiment using audio-visual McGurk stimuli produced with manual cues. The manual cue was either congruent with auditory information, lip information or with the expected fusion. We examined whether or not these experimental conditions would impact the pattern of responses differently for deaf and hearing subjects.

## Materials and methods

### Participants

Thirty-seven adults participated in the study. They were split into three groups according to their auditory status and degree of CS expertise. The first group consisted of eight deaf CS users (mean age: 18 years), hereafter referred to as the CS-deaf group. Three of them had cochlear implants and five used binaural hearing aids. Seven had been exposed to CS from the age of two to three years and the remaining one from the age of 14 years (for more details see Table 1) The second group was comprised of 14 hearing CS users (mean age: 22 years), hereafter referred to as the CS-hearing group. Two of them had close relatives that were deaf; the rest were students in speech therapy and had participated in CS training sessions. The third group consisted of 15 hearing-individuals who had never been exposed to CS (mean age: 23 years), hereafter referred to as the control hearing group.

**Table 1:** **CS-deaf group characteristics**.

**Participants**	**Age (in years)**	**Age at diagnosis**	**Age at equipment (in years)**	**Age at CS exposure (in years)**
1	17	At birth	Unknown	2
2	21	3 years	3	3
3	21	At birth	2	3
4	14	At birth	3	2
5	24	At birth	3	2
6^*^	21	At birth	5	2
7^*^	16	At birth	8	2
8^*^	17	2 years	16	14

* Indicates participants with cochlear implants.

All participants were native French speakers with normal or corrected-to-normal vision and did not have any language or cognitive disorder. In order to assess CS knowledge level, a French CS reception test was administered to all participants (TERMO). Scores groups and participants are indicated in Appendix, Table A1. The experimental protocol was approved by the ethical committee of the Faculty of Psychological Science and Education (Université Libre de Bruxelles). All participants provided informed consent, indicating their agreement to participate in study. They were informed they had the option to withdraw from the study at any time.

### Experimental material

#### Stimuli

**Figure 2:**
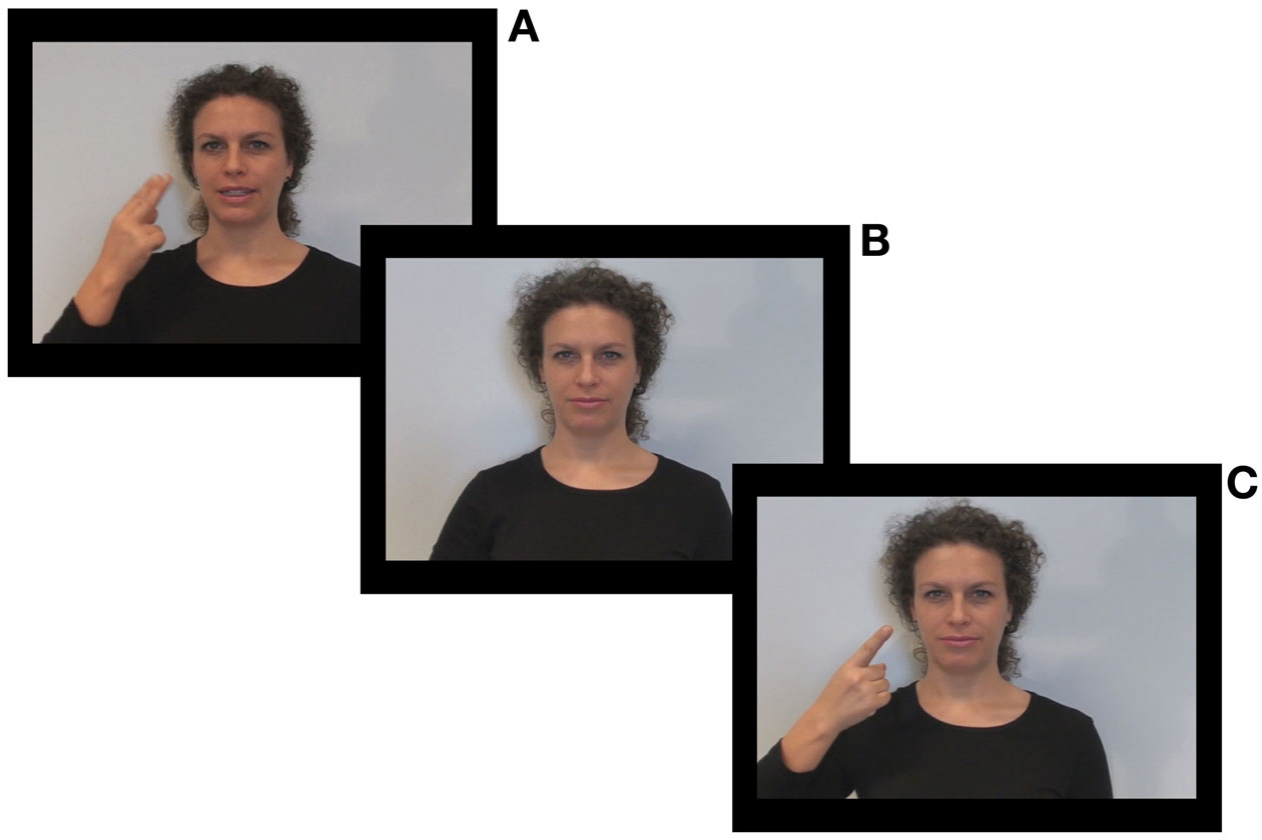
**Stimulus sample**. Video frame of lip-reading with congruent cue condition **(A)**, of audio only condition **(B)**, of audio with congruent cue condition **(C)**.

A female French speaker was videotaped while uttering CV syllables consisting of one of the /p, k, t/ consonants articulated with /a/ (Figure 2).

#### Congruent conditions

Two uni-modal and four multi-signal congruent conditions were created (see Table 2). They served as control conditions. Each stimulus from the congruent conditions was presented 6 times.

**Table 2:** **Stimulus composition of congruent control conditions**.

**Conditions**	**Stimulus 1**	**Stimulus 2**	**Stimulus 3**
Audio only	A /pa/	A /ta/	A /ka/
Lip-reading only	LR /pa/	LR /ta/	LR /ka/
Audio + CS cue	A /pa/ + CS cue coding /**p**, d, Ʒ/	A /ta/ + CS cue coding /m, **t**, f/	A /ka/ + CS cue coding /**k**, v, z/
Lip-reading + CS cue	LR /pa/ + CS cue coding /**p**, d, Ʒ/	LR /ta/ + CS cue coding /m, **t**, f/	LR /ka/ + CS cue coding /**k**, v, z/
Audio visual	A /pa + LR /pa/	A /ta/ + LR /ta/	A /ka/ + LR /ka/
AV + CS cue	A /pa/ + LR /pa/ + CS cue coding /**p**, d, Ʒ/	/	/

Because each CS cue codes several phonemes, the phoneme congruent with auditory information, or lip-reading information is indicated in bold.

#### Incongruent conditions

Stimuli were also presented in incongruent conditions. Incongruent AV syllables were created by carefully combining audio files /pa/ with non-corresponding video files /ka/ and matching their onset. Four incongruent conditions were created which consisted of McGurk stimuli (audio/pa/ and lip-reading /ka/) presented with or without manual cues (see Table 3). Each stimulus from the incongruent condition was presented 6 times.

**Table 3:** **The composition of McGurk stimuli in incongruent conditions**.

	**Auditory info**.	**Lip reading info**.	**Manual cue info**.
Baseline condition	pa	ka	/
Audio condition	pa	ka	**pa**, da, Ʒa (congruent with auditory information)
Lip-reading condition	pa	ka	**ka**, va, za (congruent with lip read information)
Fusion condition	pa	ka	ma, **ta**, fa (congruent with the expected fusion)

Because each CS cue codes several phonemes, the phoneme congruent with auditory information, or lip-read information, or the expected fusion is indicated in bold.

### Procedure

The experiment took place in a quiet room. Videos were displayed on a 17.3 inch monitor on a black background at eye level and at 70 cm from the participant's head. The audio track was presented at 65 dB SPL (deaf participants used their hearing-aids during the experiment). On each trial, participants saw a speaker's video (duration 1000 ms; see Figure 2). They were then asked to repeat aloud the perceived syllable. Their answers were transcribed by the experimenter. The experiment consisted of 120 items (16 × 6 congruent stimuli and 4 × 6 incongruent stimuli) presented in two blocks of 60 items. In each block, all conditions were mixed. Before starting, participants were shown five training items. The total duration of the experiment was approximately 30 min.

## Results

### Congruent conditions

As the groups were small (*N* < 15), we used non-parametric tests. In the congruent condition, we wanted to compare participants according to two criteria: auditory status (hearing vs. deaf) and CS abilities (CS users vs. non-CS users). Mann-Whitney tests were used to compare hearing (CS and non-CS together) with deaf groups and to compare CS users (deaf and hearing together) with the control group.

#### Audio conditions (with or without CS cue)

As illustrated in Table 4, in the Audio-Only condition, deaf and hearing-individuals had the same percentage of correct responses for the stimulus /pa/ (*U* = 91; *p* = 0.184). As it appeared that the standard deviation for the deaf group (18.2) was much higher than that of the hearing group, we analyzed individual scores of the deaf participants. Participant 2 was the only one to have a score under 83%; he obtained only 17% of correct responses. As confirmed by TERMO scores (Table 1), despite his binaural hearing aids, participant 2 had a low level of auditory recovery. When data were re-analyzed without this atypical participant, the outcome remained unchanged: Deaf and hearing-individuals had the same percentage of correct responses for the stimulus /pa/ (*U* = 91; *p* = 0.373). However, the CS-deaf group had more difficulty than the two hearing groups in identifying stimuli /ta/ (*U* = 29; *p* < 0.005) and /ka/ (*U* = 43.50; *p* < 0.005). Compared to the Audio-Only condition, the addition of cues improved the percentages of correct answers for the CS-deaf group, nonetheless the hearing groups still had more correct responses for /pa/ (*U* = 73.5; *p* < 0.05), /ta/ (*U* = 31.5; *p* < 0.001) and /ka/ (*U* = 87; *p* < 0.01)

**Table 4:** **Mean percentages of correct responses for all groups in Audio-Only and Audio + CS cue conditions**.

	**CS-deaf**		**CS-hearing**		**Control hearing**	
	**Audio only cond**.	**Audio + CS cue cond**.	**Audio only cond**.	**Audio + CS cue cond**.	**Audio only cond**.	**Audio + CS cue cond**.
/pa/	85 (18.2)	93 (12.5)	100 (0)	98 (2.4)	98 (2.1)	95 (7.1)
/ta/	62 (21.9)	70 (23.9)	100 (0)	98 (0)	100 (0)	100 (0)
/ka/	59 (29.2)	93 (9.4)	100 (0)	100 (0)	100 (0)	100 (0)

Standard deviations are indicated in parentheses.

#### Lip-reading conditions (with or without CS cue)

In the Lip-reading-Only condition, both deaf and hearing participants had similar percentages of correct responses for /pa/ (*U* = 77; *p* = 0.068), /ta/ (*U* = 157; *p* = 0.37) and /ka/ (*U* = 170.5; *p* = 0.173). The addition of cues, in comparison with the Lip-reading-Only condition, increased the percentages of correct answers for CS users (deaf and hearing). CS users had better responses than control participants for /pa/ (*U* = 98; *p* < 0.05), /ta/ (*U* = 82.5; *p* < 0.01), and /ka/ (*U* = 98.5; *p* < 0.05). Percentages of correct responses for each group are shown in Table 5.

**Table 5:** **Mean percentages of correct responses for all groups in Lip-reading-Only and Lip-reading + CS cue conditions**.

	**CS-deaf**		**CS-hearing**		**Control hearing**	
	**Lip-reading-Only cond**.	**Lip-reading + CS cue cond**.	**Lip-reading-Only cond**.	**Lip-reading + CS cue cond**.	**Lip-reading-Only cond**.	**Lip-reading + CS cue cond**.
/pa/	68 (18.8)	100 (0)	71 (18.7)	91 (9.9)	91 (10.7)	77 (17.8)
/ta/	52 (27.1)	85 (18.2)	38 (27.8)	69 (36.9)	46 (24)	38 (24.4)
/ka/	22 (14.6)	89 (15.6)	8 (11.0)	69 (22.9)	14 (13.5)	52 (24.9)

Standard deviations are indicated in parentheses.

#### Audio with lip-reading conditions (with or without CS cue)

As illustrated in Table 6, deaf and hearing-individuals obtained 100% of correct responses for the AV stimulus /pa/. However, the CS-deaf group had more difficulty than either of the two hearing groups in identifying AV stimuli /ta/ (*U* = 43.5; *p* < 0.01) and /ka/ (*U* = 43.5; *p* < 0.01). Deaf participants did not obtain 100% of correct responses for stimuli /ta/ and /ka/, because both the audio and visual information were difficult to identify (audio /ta/ 62%, audio /ka/ 59%, lip-reading /ta/ 52% and lip-reading /ka/ 22%; Tables 4, 5).

**Table 6:** **Mean percentages of correct responses for all groups in Audio + Lip-reading (LR) and Audio + LR + CS cue conditions**.

	**CS-deaf**	**CS-hearing**	**Control hearing**
Audio /pa/ + LR /pa/	100 (0)	100 (0)	100 (0)
Audio /ta/ + LR /ta/	64 (27.1)	100 (0)	100 (0)
Audio /ka/ + LR /ka/	62 (26.0)	100 (0)	100 (0)
Audio /pa/ + LR /pa/ + CS /pa/	100 (0)	100 (0)	100 (0)

Standard deviations are indicated in parentheses.

When all information (auditory, labial and manual) were presented, participants had the same percentage of correct responses for /pa/.

### Incongruent conditions

Participant responses were classified into four categories: audio (when the response was /pa/), lip-reading (when the response was /ka/), fusion (when the response was /ta/) and other. In the baseline condition, we used Mann-Whitney tests to compare hearing (CS and non-CS together) with deaf groups. In each group, the Wilcoxon test was used to compare response patterns between baseline and other experimental conditions.

#### McGurk—baseline condition (audio /pa/ + lip-reading /ka/)

As illustrated in Table 7, deaf and hearing-individuals had the same percentages of fusion (*p* = 0.39) and auditory (*p* = 0.18) responses.

**Table 7:** **Mean percentages of each kind of response (audio, lip-reading, fusion and other) for all groups in incongruent conditions**.

	**CS-deaf**	**CS-hearing**	**Control hearing**
**McGurk—Baseline condition (audio /pa/ + lip-reading /ka/)**			
Resp. audio /pa/	8 (14.6)	17 (20.5)	27 (28.9)
Resp. lip-reading /ka/	2 (3.6)	1 (2.4)	1 (2.1)
Resp. fusion /ta/	81 (24)	78 (20.7)	70 (29.3)
Other response	9 (10.4)	2 (4.3)	2 (2.1)
**McGurk—Audio condition (audio /pa/ + lip-reading /ka/ + CS cue coding /p,d,Ʒ/)**			
**Resp. audio /pa/**	**18 (19.8)**	**60 (25)**	**37 (34.8)**
Resp. lip-reading /ka/	2 (3.6)	0 (0)	1 (2.1)
Resp. fusion /ta/	20 (27.1)	21 (22.5)	57 (32.9)
Other response	60 (31.2)	18 (21.5)	5 (5.8)
**McGurk—Lip-reading condition (audio /pa/ + lip-reading /ka/ + CS cue coding /k,v,z/)**			
Resp. audio /pa/	2 (3.6)	20 (21.1)	35 (33.4)
**Resp. lip-reading /ka/**	**60 (32.8)**	**40 (27.4)**	**2 (3.9)**
Resp. fusion /ta/	25 (22.9)	33 (24.1)	61 (30.4)
Other response	13 (18.7)	6 (7.9)	2 (2.1)
**McGurk—Fusion condition (audio /pa/ + lip-reading /ka/ + CS cue coding /m,t,f/)**			
Resp. audio /pa/	0 (0)	16 (23.7)	35 (33.8)
Resp. lip-reading /ka/	0 (0)	0 (0)	1 (2.1)
**Resp. fusion /ta/**	**91 (10.4)**	**75 (28.6)**	**61 (31.1)**
Other response	9 (10.4)	9 (13.8)	3 (3.9)

Standard deviations are indicated in parentheses. Audio, lip-reading or fusion response congruent with CS cue information are indicated in bold.

#### McGurk—audio condition (audio /pa/ + lip-reading /ka/ + CS cue coding /p,d,Ʒ/)

Response patterns for each group in the McGurk-audio condition are shown in Table 7. Compared to the baseline condition, the addition of the /p, d, Ʒ/ cue reduced the percentage of fusion responses in the CS-deaf group (*p* = 0.03) in favor of other responses congruent with cue information (60% of other responses: 38% of /da/ and 19% of /Ʒa/). In the CS-hearing group, the addition of cue n°1 reduced the percentage of fusion responses (*p* = 0.001) and increased auditory responses from 17% to 60% (*p* = 0.003). In the Control hearing group, the addition of the cue had no effect on the response pattern.

#### McGurk—lip-reading condition (audio /pa/ + lip-reading /ka/ + CS cue coding /k,v,z/)

As illustrated in Table 7, the addition of the cue coding /k, v, z/ in the CS-deaf group, reduced the percentage of fusion responses (*p* = 0.02) and increased the percentage of lip-reading responses (*p* = 0.03), in comparison with the baseline condition. In addition, some participants responded with the alternative, /za/, which was congruent with cue information. In the CS-hearing group, the addition of cue n°2 also decreased fusion responses (*p* = 0.002) and increased lip-reading responses (*p* = 0.003). In the Control hearing group, the addition of cue had no effect on the response pattern.

#### McGurk—fusion condition (audio /pa/ + lip-reading /ka/ + CS cue coding /m,t,f/)

In all groups, the addition of the cue coding /m, t, f/ had no effect on response patterns (see Table 7). There was no increase of fusion responses when compared to the baseline condition.

## Discussion

The goal of the present study was to examine how manual cue information is integrated in AV speech perception. We examined whether CS receivers can combine auditory, lip and manual information to produce a unitary percept. We expected that CS would modulate the respective weights of lip-read and auditory information differently, depending on auditory status.

### Cued speech benefit

The present data confirmed previous results (Nicholls and Ling, 1982; Périer et al., 1990) indicating that the addition of congruent cues to lip-read information improved performance in CS perception for CS users (both deaf and hearing). In the CS-deaf group, the percentage of correct answers rose respectively from 47.3% in the Lip-reading-Only condition to 91.3% in the Lip-reading with Manual Cue condition, whereas it increased from 39 to 76.3% in the CS-hearing group (see Table 5). CS is therefore an efficient system to aid deaf people in perceiving speech visually. Note that for the CS-deaf group, manual cues with audio information also showed an improvement in perception. Indeed, the percentage of correct responses increased from 68.7% in the Audio-Only condition to 85.3% in the Audio with Manual Cue condition (see Table 4).

In contrast, the addition of cues decreased performance for the control group. It seems as though the CS cue served as a distractor for this group causing a disruption in responses. Their attention could have been drawn to the hand gesture, resulting in less focus on lip-read information. Compared to the Lip-reading-Only condition, the addition of cues decreased their percentages of correct responses, despite showing no significant effect. Furthermore, in the McGurk conditions with manual cues, the presence of hand information possibly unbound audio and visual information. Being more attracted to irrelevant hand information than by lip information, participants tended to not integrate AV information, resulting in fewer fusion responses and favoring auditory responses.

### Audio-visual speech integration in deaf

Our results showed that deaf people with cochlear implants or binaural hearing aids can merge auditory and lip-reading information into a unified percept just as hearing-individuals do. In the baseline condition (audio /pa/ + lip-reading /ka/), percentages of fusion responses were high and similar for both hearing and deaf groups (74 and 81% respectively, Table 7). Contrary to previous studies (Leybaert and Colin, 2007; Desai et al., 2008; Rouger et al., 2008), deaf individuals did not tend to report more responses based on visual information than hearing-participants. One explanation might be that deaf and hearing-individuals both exhibited comparable levels of performance in uni-modal conditions: percentages for identification of the auditory syllable /pa/ and the lip-reading syllable /ka/ did not differ between neither deaf nor hearing groups.

### Manual cue effect on audio-visual speech integration

In the case of incongruent auditory and visual information (audio /pa/ and lip-reading /ka/), the addition of manual cues that were incongruent with the expected fusion response impacted the pattern of responses. For both deaf- and hearing-CS users, the proportion of fusion responses decreased. The CS system therefore has an effect on AV integration processes. In the case of congruency between manual cues and expected fusion, the CS system supports illusory perception. However, for all groups the percentage of fusion did not increase. One explanation might be that the proportion of fusion responses in the baseline condition was already fairly high in deaf and hearing groups (81 and 74%, respectively Table 7).

Whereas manual cues decreased fusion responses in both hearing- and deaf-CS users, their effect on other responses depended on auditory status. Indeed, the addition of manual cues congruent with auditory information (but not with lip-read information) increased only audio responses for /pa/ in the CS-hearing group but not in the CS-deaf group. In this latter group, fusion responses decreased in favor of other responses, congruent with the manual cue coding /p, d, Ʒ/ (i.e., response /da/ or / Ʒa/). Thus, despite their good performance in the Audio-Only condition (85%), CS-deaf users seemed more confident with visual information (such as lip-read or manual cues). They were unable ignore lip-read information and relied more heavily on such information than on auditory.

The addition of manual cues congruent with lip-read information increased lip-reading responses in both groups. These results suggest that deaf- and hearing-CS users are capable of ignoring auditory information when such information is contradicted by lip-reading or manual cues. As the CS system is not necessarily used with auditory information, ignoring auditory information could be easier.

### Auditory status effect or auditory abilities effect?

**Figure 3:**
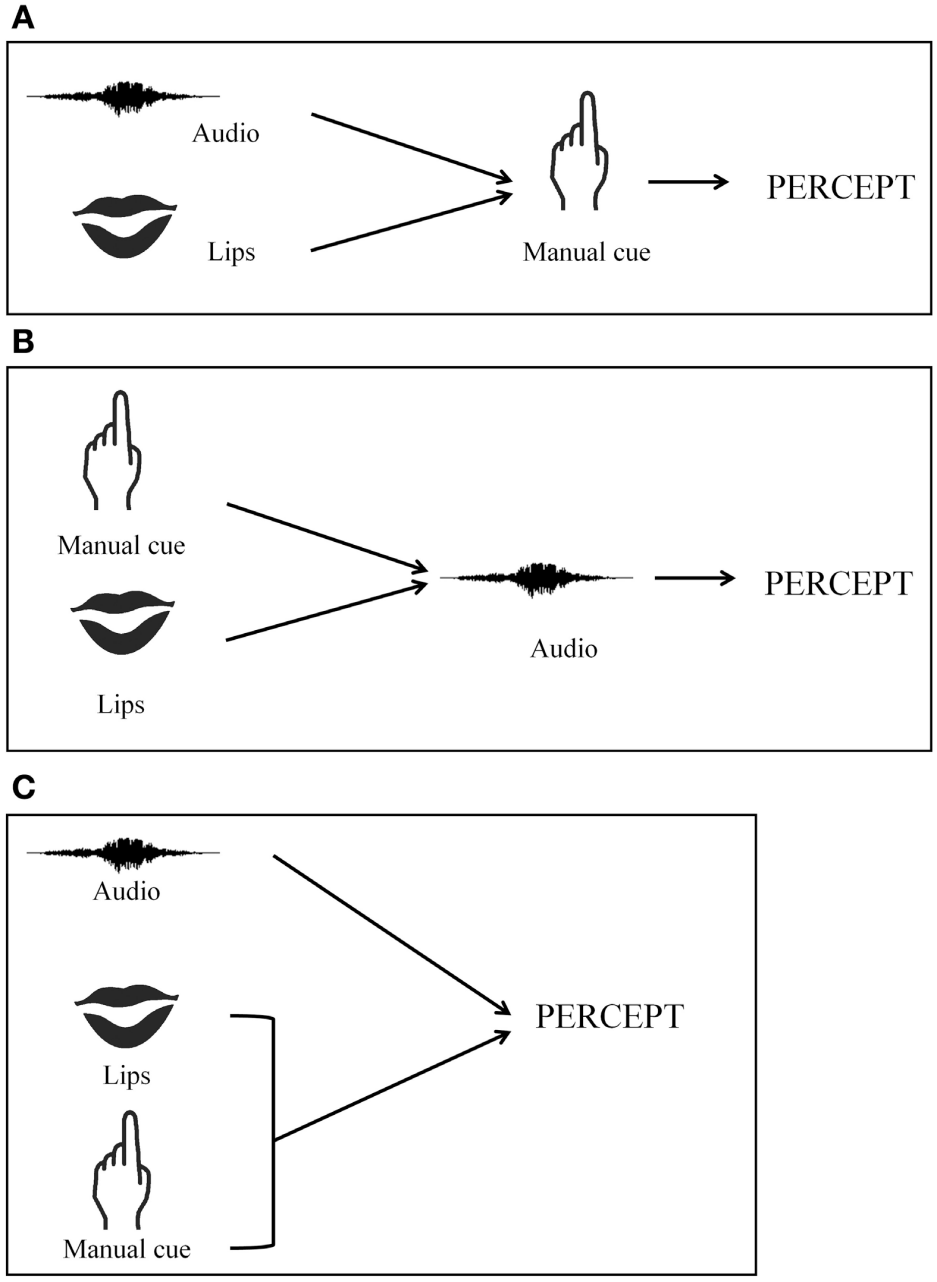
**CS perception models**. **(A)** Sequential model with late integration of manual cue; **(B)** Sequential model with early integration of manual cue; **(C)** Simultaneous model with early integration of manual cue.

Deaf-CS users' multimodal speech perceptions differ from that of hearing CS-users. Our results have shown that the addition of manual cues congruent with auditory information impacts the speech perception of deaf and hearing-individuals differently. Perception for deaf individuals relies more on visual information (lip-reading and manual cues); whereas perception in hearing-CS users relies more on auditory information. This suggests that the processing of CS information is modulated by auditory status. We have envisioned two speech perception models in order to explain these results. As it is illustrated in Figure 3A, hearing-CS receptors integrate auditory and labial information first, before determining whether manual cues are helpful in assembling a coherent percept. While manual cues might precede labial and auditory stimuli (Attina et al., 2004), hearing-individuals are more prone to ignore manual information and give more auditory responses in lieu of incongruent AV stimuli. CS perception remains less natural for hearing-individuals than for deaf. In the second model (see Figure 3B), deaf-CS receptors first integrate manual and lip information before taking auditory information into account. Thus, deaf-CS users cannot ignore manual information, resulting in less auditory responses. However, in our experiment, the deaf-CS user group was too small of a sample to be split into two groups according to the participants' auditory recuperation. We were therefore not able to examine the effect of auditory recuperation on the nature of integration processes. Auditory status and auditory abilities were thus confounded, which renders our interpretation fragile.

Therefore, in a new study (Bayard et al., in preparation), we investigated whether auditory status or auditory abilities impact audio-lip-read-manual integration in speech perception by testing a larger sample of deaf individuals whom of which were fitted with cochlear implants. Our first collection of data suggests an effect of auditory ability. Deaf individuals with good auditory ability had the same pattern response as their hearing-counterparts. Thus, for hearing- and deaf individuals with good auditory speech perception abilities, speech perception may first involve an integration between auditory and lip-read information. The merged percept then could be impacted by manual information when such information is delivered (Figure 3A). For deaf individuals with low auditory ability, labial and manual information could be initially merged, and auditory information would be taken into account subsequently (Figure 3B).

A number of other studies have revealed an impact of CI proficiency on AV speech integration. For example, Landry et al. (2012), compared three groups in a lip-reading task: proficient CI group, non-proficient CI group and normally-hearing group. Participants had to report visual speech stimulus presented in four conditions: visual only condition, AV speech condition, AV white noise condition, and AV reverse speech condition. Participants were informed that all auditory inputs were incongruent with the visual stimulus. Results showed that the presentation of auditory speech stimuli significantly impaired lip-reading performance only in proficient CI users and the normally-hearing group. Non-proficient CI users were not affected by auditory distractors, suggesting that such distraction was ignored due to their poor auditory ability. Huyse et al. (2013) showed that patterns of auditory, visual, and fusion responses to McGurk audio-visual stimuli are relative to CI proficiency. CI children who are AO− seemed to rely more on vision and CI children who are AO+ seemed to rely more on auditory information. Although these studies analyzed AV perception without cues, they reinforce our proposition that we should distinguish AO+ and AO− profiles in future studies of speech perception in participants with CI and CS.

### Integration of the CS component in speech perception models

Many AV integration studies on hearing-individuals have attempted to determine how and when integration takes place. More specifically, the issue of whether integration is early (before phonetic categorization) or late (after phonetic categorization) has been a topic of empirical and theoretical research. A number of speech perception and AV integration models have been proposed. Among such designs, the “Fuzzy logical model of perception” (FLMP; Massaro, 1987) postulates the existence of two stages in AV speech perception. The first stage is uni-modal processing. Auditory and visual features are assessed and compared to prototypes stored in memory. Comparison is based on a continued value scale and is independent in each modality. The second stage is bi-modal. Values of each feature are integrated in order to determine the degree of global adequacy of sensory input with each prototype in memory. The prototype that is the most consistent with the features extracted during the uni-modal assessment will be the percept heard. One important issue in this model is the fact that the influence of each source of information depends on its ambiguity. The more ambiguous the source, the less it influences perception. In addition, according to FLMP, all individuals integrate AV information optimally. In this way, all differences in the percept have to be explained by differences within the initial, uni-modal, stage.

The “Weight fuzzy logical model of perception” (WFLMP) is an interesting adaptation of FLMP (Schwartz, 2010). In WFLMP, inter-individual differences are taken into account. For each individual, specific weights may be allocated to each modality (visual and auditory). In WFLMP, differences in percept could be explained both by differences in uni-modal perception as well as by differences in integrative processing. As previous studies on speech perception in deaf-CI users have shown inter-individual differences (Landry et al., 2012; Huyse et al., 2013), the WFLMP seems to be more adapted than the FLMP in explaining such differences in perception. Recently, Huyse et al. (2013) conducted a study on speech perception in CI users and normally-hearing children. They tested the robustness of bias toward the visual modality in McGurk stimuli perception in CI users. For that reason, they designed an experiment in which the performances were compared in a “visual clear” condition and a “visual reduction” condition, in which the visual speech cues were degraded. Results showed that “visual reduction” had increased the number of auditory-based responses to McGurk stimuli, in normally-hearing as well as CI children (whose perception is generally dominated by vision). The authors used both FMLP and WFLMP to determine whether the differences in response patterns between “visual reduction” and “visual clear” conditions occurred at the uni-modal processing stage or at the integration stage. The FLMP model better fits the data in the “visual reduction” condition when an additional weight is applied to the auditory modality. The degradation of visual information seems to have an impact on speech perception not only at the uni-modal stage of processing but at the integrative processing level, as well. Thus, WFLMP seems to be a relevant model to explain AV speech perception in CI-users.

In the context of CI + CS perception, a third source of information is added: manual cue information. How is manual information processed in the WFMLP framework? We foresee three possibilities. According to a *first hypothesis*, the two types of visual information (manual cue and lip-read information) are processed in parallel and constitute the uni-modal, visual signal (Figure 3C). The influence of visual information (labial and manual) could be more important in both the uni-modal and integration stages of processing, in comparison to what occurs in classical AV integration. According to *the second hypothesis*, AV integration occurs as Schwartz described in WFLMP, and the manual cue information is merged with the AV percept later in integrative processing (Figure 3A). According to a *third hypothesis*, the labial- and manual-visual information are merged first, and auditory information is taken into account later (Figure 3B).

Currently, our studies have not allowed us to choose between these three hypotheses. It is clear that manual cue could impact AV integration. However, our behavioral data are not sufficient to determine whether this impact occurs early (as in the first hypothesis) or later (as in the second hypothesis). Furthermore, we have learned that deaf participants are capable of ignoring auditory cues, whereas they cannot ignore labial or manual information. Thus, for future studies, we aim to analyze more precisely the effect of auditory efficiency on speech perception, using data to confront our hypotheses.

In natural speech (without CS), humans speak and spontaneously produce gestures to support what they are saying. Analysis of speech and symbolic gesture production in adults suggest that both “are coded as a unique signal by a unique communication system” (Bernadis and Gentilucci, 2006). In addition, gestures play a crucial role in language development and a co-development of speech and signs exists (for a review see Capone and McGregor, 2004). Thus gesturing seems to be a genuine component of multi-modal communication. CS cues are created specifically for communication. Due to this privileged link between gestures and language, it is probable that these cues are naturally integrated into multi-modal communication. As shown by our data, it is difficult to ignore information provided by a cue.

## Conclusion

Speech perception is a multimodal process in which different kinds of information are likely to be merged: naturally and relevant information (provided by lip-reading and audition), naturally but irrelevant information (like in audio-aerotactile integration), or non-natural but relevant information (such as CS cues).

Findings from our work also suggest that the integration of different types of information (e.g., audition, lip-reading, manual cues) related to a common source (i.e., the production of a speech signal) is a flexible process that depends on the informational content from the different sources of information, as well as on the auditory status and hearing proficiency of the participants.

### Conflict of interest statement

The authors declare that the research was conducted in the absence of any commercial or financial relationships that could be construed as a potential conflict of interest.

## Appendix

**Table A1:** **TERMO scores by group and participant for Audio-Only, Visual-Only, AV, and Visual with CS (V + CS) cue conditions**.

	**Audio**	**Visual**	**AV**	**V + CS**	**Audio gain**	**CS gain**
DEAF CS	60.38 (22.30)	35.88 (11.61)	77.00 (18.40)	94.75 (5.01)	41.13 (22.34)	58.89 (12.29)
1	82	24	82	94	58	70
2	12	29	41	100	12	71
3	71	35	94	88	59	53
4	59	35	88	100	53	65
5	65	47	94	88	47	41
6^*^	71	29	76	94	47	65
7^*^	47	59	59	100	0	41
8^*^	76	29	82	94	53	65
HEARING CS	100 (0.00)	38.14 (9.92)	100 (0.00)	88.43 (3.69)	61.86 (9.2)	50.29 (9.92)
1	100	47	100	88	53	41
2	100	41	100	88	59	47
3	100	41	100	88	59	47
4	100	24	100	82	76	58
5	100	29	100	88	71	59
6	100	41	100	82	59	41
7	100	35	100	88	65	53
8	100	59	100	94	41	35
9	100	18	100	94	82	76
10	100	35	100	88	65	53
11	100	41	100	94	59	53
12	100	41	100	88	59	47
13	100	41	100	88	59	47
14	100	41	100	88	59	47
HEARING CONTROL	99.60 (1.55)	36.27 (8.39)	100 (0.00)	42.33 (10.91)	63.73 (8.39)	6.07 (12.70)
1	100	41	100	47	59	6
2	100	47	100	47	53	0
3	100	41	100	47	59	6
4	100	29	100	35	71	6
5	100	47	100	59	53	12
6	100	35	100	35	65	0
7	100	18	100	47	82	29
8	100	29	100	53	71	24
9	100	35	100	41	65	6
10	100	41	100	53	59	12
11	100	29	100	53	71	24
12	100	29	100	24	71	−5
13	94	41	100	24	59	−17
14	100	35	100	29	65	−6
15	100	47	100	41	53	−6

*Standard deviations are indicated in parentheses*.

* Indicates participants with cochlear implants.
